# The association between serum lipid profile and the prostate cancer risk and aggressiveness

**DOI:** 10.3389/fonc.2023.1113226

**Published:** 2023-05-15

**Authors:** Jungyo Suh, Teak Jun Shin, Dalsan You, In Gab Jeong, Jun Hyuk Hong, Choung-Soo Kim, Hanjong Ahn

**Affiliations:** ^1^ Department of Urology, University of Ulsan College of Medicine, Asan Medical Center, Seoul, Republic of Korea; ^2^ Department of Urology, School of Medicine, Keimyung University, Daegu, Republic of Korea; ^3^ Department of Urology, Ewha Womans Medical Center, Seoul, Republic of Korea

**Keywords:** cancer risk, lipid profile, metabolic syndrome, prostate cancer, triglycerides

## Abstract

**Purpose:**

This study aims to evaluate the association of serum lipid profile on prostate cancer (PC) risk and aggressiveness.

**Methods:**

Men who underwent prostate biopsy between January 2005 and December 2015 were retrospectively analyzed. The association between lipid profile and the risk, stage, and Gleason grade group (GG) of the PC were investigated. Sensitivity analysis was conducted using univariate and multivariate quantile analysis for lipide profile on the risk and stage of PC.

**Results:**

Of the 1740 study populations, 720 men (41.4%) were diagnosed as PC. From multivariate logistic regression analysis, age, prostate specific antigen, triglyceride (odds ratio (OR):1.05, confidence interval (CI):1.03-1.07, p-value<0.001) significantly increased PC risk, while total cholesterol (OR:0.96, CI:0.92-0.99, p-value=0.011) significantly decreased the PC risk. The increase of serum triglyceride increased the risk of both of locally advanced (OR:1.03, CI:1.00-1.07, p-value=0.025) and metastatic PC (OR:1.14, CI:1.04-1.25, p-value=0.004). The increase of serum triglyceride increased the risk of GG2-3 (OR:1.03, CI:1.00-1.06, p-value=0.027) and GG4-5 (OR:1.04, CI:1.01-1.08, p-value=0.027). Univariate quartile analysis founded serum triglyceride increasing risk of locally advanced disease than organ confined disease. (OR: 1.00, 1.25, 2.04, 4.57 for 1^st^, 2^nd^, 3^rd^ and 4^th^ quartile, p-value<0.001). Adjusted multivariate quartile analysis confirmed statistically significant increasing PC risk of triglyceride (OR: 1.00, 1.25, 2.04, 4.57 for 1^st^, 2^nd^, 3^rd^ and 4^th^ quartile, p-value<0.001).

**Conclusions:**

This study findings suggested increased in triglyceride level increased the risk PC. Increased in triglyceride level also associated with aggressive presentation of PC, with higher stage and GG.

## 1 Introduction

Prostate cancer (PC) is the second most common malignancy in men and the second leading cause of cancer death worldwide (1). The age-standardised incidence of PC can differ 25-fold depending on geographic location; generally higher in Western compared with in Asian countries (2). There is an increased incidence of PC in Asian immigrants to Western countries (3) and temporal changes after adaptation to a westernized lifestyle in the northeast Asian population over the last decades (4, 5) suggest that an environmental factor highly influences the PC risk.

Dyslipidaemia is part of metabolic syndrome, characterized by an alteration of the plasma lipid profile, including LDL, HDL, and triglyceride levels. Both of genetic and environmental factors influence serum lipid levels (6, 7). Metabolic syndrome is another most common medical problem and is continuously increase in world-wide (8). The increasing incidence of both of PC and the metabolic syndrome suggest potential linkage between two diseases (8). While dyslipidaemia has been shown to be associated with PC risk in some *in vivo* and *in vitro* studies (9–12), epidemiologic and clinical studies have yielded conflicting results (13–16). Understanding the association between the lipid profile and prostate cancer risk is important because the lipid profile is considered to be a potentially modifiable risk factor.

Recent evidence suggests that metabolic syndrome might be associated with prostate cancer risk, aggressive features, and recurrence after treatment (15, 17). However, there is still debate surrounding the association between specific lipid profile and PC risk. This study aims to evaluate the association between the serum lipid profile and PC risk, aggressiveness, and staging.

## 2 Materials and methods

### 2.1 Study design and population

This study was approved by the Institutional Review Board (*IRB No. 2022-1479*) of the Asan Medical Center. The medical records of men who underwent a transrectal ultrasound (TRUS)-guided prostate biopsy at our institution between January 2005 and December 2015 were retrospectively reviewed. Prostate biopsies were conducted on men with a serum PSA level ≥ 4.0 ng/mL before 2009 and ≥ 3.0 ng/mL after that or if prostate cancer was clinically suspected. Among those who underwent a prostate biopsy, patients with a measured serum glucose and lipid profile, including the levels of serum total cholesterol, low-density level cholesterol (LDL, mg/dL), high-density level cholesterol (HDL, mg/dL), and triglyceride (mg/dL) in a fasting state within one year before prostate biopsy were enrolled. Quantification of each component of lipid profile measured by Beckman Coulter AU 5800 chemistry analyzer (Beckman Coulter, Brea, CA) (18). Age, body mass index (BMI), hypertension and diabetes were measured at the time of the biopsy. HbA1c was included in the values measured within three months before the biopsy, and statin co-medication was included in the analyses if the statin was taken for more than one month before the biopsy.

### 2.2 Statistical analysis

Continuous variables were described by mean ± standard deviation, and discrete variables were described by number and frequency. Differences in demographic, clinical, and pathological factors between men with non-prostate cancer versus prostate cancer were examined using t-tests and Chi-squared (χ2) tests for continuous and categorical variables, respectively. Differences in the variables between Gleason grade (GG) and clinical stage were analysed with a one-way ANOVA test. Multivariate logistic regression models were used to assess the association between the PC risk and collected variables, including lipid profile. In addition, we analysed the association between PC aggressiveness and collected variables, by sub-group analysis of the Gleason grade group (GG; 1, 2-3 and 4-5) (19) and clinical stage (organ confined, locally advanced and metastatic prostate cancer). Locally advanced disease was defined as clinical stage is above T3 or suspected regional lymph node metastasis. Metastatic disease was defined as presence of distant or nonregional lymph node metastasis (20). The variables that showed p-value < 0.05 in the univariate analysis were selected for multivariate analysis. Finally, sensitivity analysis was conducted using adjusted univariate and multivariate quantile analysis for each subset of the lipid profile on the risk and stage of PC. All statistical analyses were done using SPSS version 21.0 (IBM, Armonk, NY, USA), and the significance level was 0.05 or less.

## 3 Results

### 3.1 Patient characteristics

Of the 1740 men who underwent prostate biopsy, 720 men (41.4%) were diagnosed with PC. Among the PC patients 34.3% (247/720), 36.4% (262/720), 29.3% (211/720) were GG 1, GG2-3 and GG 4-5. Most of patients (78.8%, 567/720) defined as localized disease and 15.5% (112/720) was locally advanced disease. Only 5.7% (41/720) were metastatic disease at the time of diagnosis. The patients who were diagnosed as PC had significantly higher age (66.3 vs. 60.1 years, p-value < 0.001), PSA level (32.9 vs. 4.7 ng/mL, p-value = 0.024) than non-PC population. The diabetes mellitus (23.2% vs. 12.4%, p-value < 0.001) and hypertension (50.0% vs. 35.5%, p-value < 0.001) were more frequent in PC patients. Statin comedication (12.0% vs. 9.7%) and BMI (24.5 vs 24.3, p-value = 0.174) was not significantly difference between PC and non-PC groups. For lipid profile, total cholesterol (178.0 vs 187.9 mg/dL, p-value < 0.001), LDL (111.7 vs. 119.6 mg/dL, p-value < 0.001) and HDL (49.8 vs. 52.0 mg/dL, p-value < 0.001) level were significantly lower and triglyceride level (141.0 vs. 119.1 mg/dL, p-value < 0.001) were significantly higher in PC patients than non-PC group. Detailed characteristics are described in **Table 1**.

**Table 1:** General characteristics between prostate cancer patients and non-cancer patients before biopsy.

	Total number of patients	Non-prostate cancer	Prostate cancer	*p*-value
Number of patients	1740	1020	720	
Patient characteristics				
Age, mean ± SD, (years)	1740	60.1 ± 8.4	66.3 ± 8.0	< 0.001
BMI, mean ± SD, (Kg/m^2^)	1740	24.3 ± 2.5	24.5 ± 2.8	0.174
HTN, number (%)	1740	362 (35.5)	360 (50.0)	< 0.001
Diabetes mellitus, number (%)	1740	126 (12.4)	167(23.2)	< 0.001
Serum glucose, mean ± SD, (mg/dL)	1740	102.5 ± 18.2	110.8 ± 26.4	< 0.001
HbA1c, mean ± SD	1552	5.7 ± 0.7	6.1 ± 1.0	< 0.001
Statin co-medication, number (%)	1740	99 (9.7)	86 (12.0)	0.141
PSA, mean ± SD, ng/mL	1740	4.7 ± 2.8	32.9 ± 335.6	0.024
Lipid profile				
Cholesterol, mean ± SD, mg/dL	1740	187.9 ± 34.2	178.0 ± 36.1	< 0.001
LDL, mean ± SD, mg/dL	1740	119.6 ± 29.9	111.7 ± 31.0	< 0.001
HDL, mean ± SD, mg/dL	1740	52.0 ± 12.6	49.8 ± 13.2	< 0.001
Triglyceride, mean ± SD, mg/dL	1740	119.1 ± 61.8	141.0 ± 73.1	< 0.001

SD, standard deviation.

### 3.2 Lipid profile and prostate cancer risk

**Figure 1:**
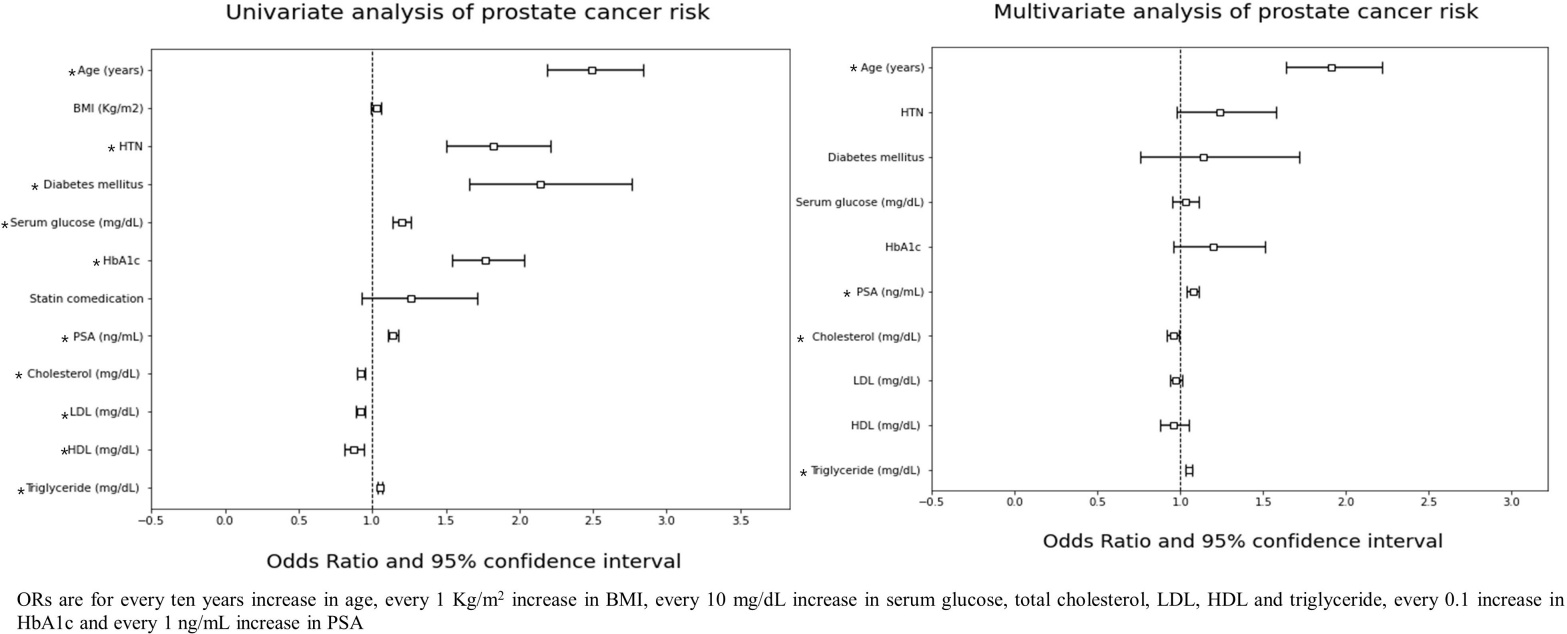
ORs are for every ten years increase in age, every 1 Kg/m^2^ increase in BMI, every 10 mg/dL increase in serum glucose, total cholesterol, LDL, HDL and triglyceride, every 0.1 increase in HbA1c and every 1 ng/mL increase in PSA.

In univariate analysis, age, hypertension, diabetes mellitus, serum glucose level, HbA1c, PSA level, and all lipid profile components were significantly associated with PC risk. The multivariate analysis showed that age {Odds ratio (OR): 1.91, 95% confidence interval (CI): 1.64-2.22, p-value < 0.001}, PSA (OR: 1.08, 95% CI: 1.04-1.11, p-value < 0.001), total cholesterol (OR: 0.96, 95% CI: 0.92-0.99, p-value = 0.011) and triglyceride level (OR: 1.05, 95% CI: 1.03-1.07, p-value < 0.001) were associated with PC risk (**Figure 1**). In the adjusted multivariate quartile analysis of PC risk and the lipid profile, statistically significant trends of increasing odds following increasing triglyceride level (p-trend < 0.001) were found, however not in total cholesterol level (p-trend =0.243) (**Supplementary Table 1**).

### 3.3 Lipid profile and prostate cancer aggressiveness: gleason grade group

Age, hypertension, diabetes mellitus, serum glucose level, HbA1c, PSA level, total cholesterol, HDL, and triglycerides were significantly different concerning GG (**Supplementary Table 2**). From univariate and multivariable analysis, age, PSA level, and triglyceride level were significantly associated with a higher GG. (**Table 2**) However, in the univariate quartile analysis of GG and the lipid profile, a statistically significant trend of odds following increasing triglyceride levels in both GG 2-3 (p-trend = 0.646) and GG 4-5 (p-trend = 0.136) (**Supplementary Table 3**) could not be found.

**Table 2:** Logistic regression analysis for pathologic Gleason grade group (GG) in prostate cancer patients.

		Univariate				Multivariate			
	GG 1 Reference	GG 2-3 OR (95% CI)	p-value	GG 4-5 OR (95% CI)	p-value	GG 2-3 OR (95% CI)	p-value	GG 4-5 OR (95% CI)	p-value
Age (years) *	1.00	1.51 (1.19–1.90)	0.001	1.94 (1.53–2.47)	<0.001	1.43 (1.12–1.82)	0.004	1.52 (1.10–2.11)	0.012
BMI (Kg/m^2^) *	1.00	0.97 (0.91–1.04)	0.415	1.00 (0.94–1.06)	0.957				
HTN	1.00	1.17 (0.83–1.66)	0.376	1.65 (1.14–2.39)	0.008			1.29 (0.79–2.11)	0.313
Diabetes mellitus	1.00	1.13 (0.73–1.75)	0.580	1.89 (1.23–2.92)	0.004			1.22 (0.55–2.70)	0.632
Serum glucose (mg/dL) *	1.00	1.02 (0.95–1.10)	0.558	1.14 (1.06–1.23)	0.001			1.05 (0.90–1.22)	0.554
HbA1c*	1.00	0.93 (0.74–1.15)	0.482	1.35 (1.10–1.65)	0.004			0.90 (0.59–1.36)	0.604
Statin comedication	1.00	1.06 (0.60–1.85)	0.844	1.47 (0.84–2.57)	0.174				
PSA*	1.00	1.06 (1.02–1.11)	0.009	1.15 (1.10–1.21)	<0.001	1.04 (1.00–1.09)	0.046	1.10 (1.04–1.16)	0.001
Lipid profile									
Cholesterol (mg/dL) *	1.00	0.98 (0.93–1.03)	0.354	0.94 (0.89–0.99)	0.014			0.94 (0.87–1.01)	0.091
LDL (mg/dL) *	1.00	0.99 (0.94–1.04)	0.673	0.94 (0.88–1.00)	0.041			0.97 (0.89–1.06)	0.465
HDL (mg/dL) *	1.00	0.89 (0.78–1.01)	0.077	0.83 (0.72–0.95)	0.008			0.84 (0.69–1.02)	0.073
Triglyceride (mg/dL) *	1.00	1.03 (1.00–1.06)	0.029	1.04 (1.01–1.07)	0.009	1.03 (1.00–1.06)	0.027	1.04 (1.01–1.08)	0.027

*ORs are for every ten years increase in age, every 1 Kg/m^2^ increase in BMI, every 10 mg/dL increase in serum glucose, total cholesterol, LDL, HDL and triglyceride, every 0.1 increase in HbA1c and every 1 ng/mL increase in PSA.

### 3.4 Lipid profile and prostate cancer aggressiveness: clinical stage

Age, hypertension, diabetes mellitus, serum glucose level, HbA1c, PSA level, HDL, and triglycerides differed significantly between organ-confined, locally advanced, and metastatic stages (**Supplementary Table 4**). From univariate and multivariable analysis, PSA, HDL, and triglyceride levels were significantly associated with locally advanced disease and age. PSA and triglyceride levels were significantly associated with metastatic disease (**Table 3**). In the univariate quartile analysis of the clinical stage and the lipid profile, a statistically significant odds trend following increasing triglyceride levels was found in locally advanced disease (p-trend = 0.003) but not in metastatic disease (p-trend = 0.119) (**Supplementary Table 3**).

**Table 3:** Logistic regression analysis for the clinical stage in prostate cancer patients.

		Univariate				Multivariate			
	Organ confined Reference	Locally advanced OR (95% CI)	p-value	Metastatic OR (95% CI)	p-value	Locally advanced OR (95% CI)	p-value	Metastatic OR (95% CI)	p-value
Age (years) *	1.00	1.56 (1.19–2.03)	0.001	2.25 (1.49–3.41)	<0.001	1.30 (0.97–1.74)	0.084	2.88 (1.26–6.56)	0.012
BMI (Kg/m^2^) *	1.00	1.08 (1.00–1.16)	0.039	0.93 (0.83–1.05)	0.245	1.06 (0.97–1.15)	0.181		
HTN	1.00	1.30 (0.86–1.95)	0.210	2.09 (1.07–4.07)	0.030			4.62 (0.94–22.85)	0.060
Diabetes mellitus	1.00	1.43 (0.90–2.26)	0.131	2.38 (1.23–4.61)	0.010			1.38 (0.24–7.83)	0.717
Serum glucose (mg/dL) *	1.00	1.08 (1.01–1.16)	0.034	1.12 (1.03–1.22)	0.010	1.03 (0.95–1.12)	0.433	0.81 (0.61–1.07)	0.143
HbA1c*	1.00	1.25 (0.99–1.59)	0.061	1.72 (1.31–2.26)	<0.001			1.61 (0.76–3.43)	0.217
Statin comedication	1.00	1.32 (0.73–2.39)	0.352	1.35 (0.55–3.33)	0.518				
PSA*	1.00	1.06 (1.04–1.08)	<0.001	1.06 (1.04–1.09)	<0.001	1.06 (1.04–1.08)	<0.001	1.07 (1.04–1.09)	<0.001
Lipid profile									
Cholesterol (mg/dL) *	1.00	0.96 (0.91–1.02)	0.188	0.92 (0.84–1.01)	0.082				
LDL (mg/dL) *	1.00	0.98 (0.92–1.05)	0.518	0.91 (0.82–1.02)	0.097				
HDL (mg/dL) *	1.00	0.68 (0.57–0.82)	<0.001	0.64 (0.48–0.85)	0.002	0.72 (0.58–0.88)	0.001	0.70 (0.43–1.15)	0.161
Triglyceride (mg/dL) *	1.00	1.05 (1.03–1.08)	<0.001	1.04 (1.00–1.09)	0.034	1.04 (1.00–1.07)	0.025	1.14 (1.04–1.25)	0.004

*ORs are for every ten years increase in age, every 1 Kg/m^2^ increase in BMI, every 10 mg/dL increase in serum glucose, total cholesterol, LDL, HDL and triglyceride, every 0.1 increase in HbA1c and every 1 ng/mL increase in PSA.

## 4 Discussion

In the present study, we found that the lipid profile components were associated with PC risk and aggressiveness (both in higher GG and advanced clinical stage). In the multivariate analysis, PC risk was significantly associated with age, PSA, and lipid profile (total cholesterol and triglyceride level). The higher GG was significantly associated with age, PSA, and triglyceride levels. Locally advanced disease was significantly associated with a higher PSA, triglyceride level, and lower HDL level. Metastatic disease was significantly associated with older age, higher PSA, and triglyceride levels. From univariate and multivariate quartile analysis, only statistically significant trends of increasing odds were found in PC risk and locally advanced disease when assessed with regard to triglyceride level.

Prostate cancer tumorigenesis and proliferation directly or indirectly affect lipid metabolism (10). The increased intake of fatty acids is required for the rapid proliferation of prostate cancer; thus, *de novo* and alternative lipogenesis pathways are enhanced (9). Accumulation of intra tumoral lipid droplets due to PC cells *via de novo* fatty acid synthesis, gave higher survival change during nutrition depletion conditions, such as progression or metastasis (21). Moreover, prostate cancer highly depends on cholesterol-derived steroid hormones, including androgens (11). In some *in vitro* studies, hypercholesterolaemia has led to prostate tumour growth, volume increase, and metastasis, and cholesterol is an important factor controlling the signal transduction of PC cells (12, 22). Some *In vivo* studies also supported correlation of lipid profile and PC progression. Inhibition of fatty acid synthesis in metastatic castration resistance PC xenograft and organoid model, reduced tumor growth by downregulate androgen receptor pathways (23). David et al. (24) reported high fat diet reprogramed PC metabolism and accelerate disease progression, through mimicking MYC overexpression in the mouse model. Thus, alteration of the lipid profile might be associated with an increased PC risk.

Despite *in vivo* and *in vitro* evidence of a positive correlation between dyslipidaemia and PC risk, epidemiologic and clinical studies show conflicting results (13–16). For example, the Swedish Apolipoprotein MOrtality RISk (AMORIS) (14) and Austrian cohort studies (13) found evidence of lipid metabolism being associated with PC risk. However, Liu et al. reviewed a large prospective cohorts and found that total blood cholesterol, HDL, and LDL levels were not associated with PC risk and high-grade PC risk (16). Unfortunately, this meta-analysis does not assess the association between triglyceride level and PC. Prostate cancer association group to investigate cancer-associated alterations in the genome (PRACTICAL) consortium underwent mendelian randomization study to reveal causal influence of lipid profile to PC risk (25). PRACTICAL consortium reported total cholesterol level does not alter PC risk, however they founded weak association of high LDL and triglyceride on PC. Owing to the complex manifestation of LDL, triglyceride, and HDL among the lipid profiles and the effect of statin comedication on prostate cancer appear complicated, it is necessary to analyze the separation of each component of lipid profile.

In the present study, the higher triglyceride level shows a robust correlation with PC risk, advanced stage, and grades. The first (14) and extended (26) results of the AMORIS cohort are similar to our study. The AMORIS study’s strength in the extensive review of the glucose and lipid profile (14). A primitive analysis shows no relation between the triglyceride level and the PC risk. But when the glucose level was controlled for, the positive correlation between high triglyceride and PC risk in high glycaemic populations was revealed. Similar to our result, the follow-up study to AMORIS showed a positive correlation between high triglyceride levels and PC aggressiveness and severity (26). AMORIS study result has limitation on lack of information of patients diabetes status and history of statin or metformin co-medication, which can provide misclassification of patients lipid profile status (26). However, our study has the strength of complete patient data, as we collected information on all patients’ diabetes status and statin-medication status. Although metformin co-medication status was not collected in this study, we collected 87.2% (1552/1780) of patients’ HbA1c status, which reflects their recent several months of diabetes control status. This allowed us to evaluate the potential impact of diabetes on the association between lipid profiles and PC risk and aggressiveness.

Several pieces of background evidence were established supporting the positive correlation between triglycerides and PC risk and aggressiveness. Several studies have reported that triglyceride-rich residues cause carcinogenesis by cell signalling pathways such as MEK/ERK and Akt pathways associated with cell growth, cell proliferation, apoptosis and lipid biosynthesis (27, 28). High triglycerides are also associated with the development of insulin resistance and an increase in insulin-like growth factor-1, as well as increased reactive oxygen species and oxidative stress, all of which are associated with PC (27, 29) The *in vitro* studies support our results, and we emphasize an intense relationship between triglycerides and PC risk and aggressiveness.

Our study had some limitations. First, due to the retrospective nature of this study, there are inevitable risk of selection bias. The staging and GG in this study based on prostate biopsy result and clinical images, thus there are disparity between clinical and pathologic stage. As a clinical observational study, the background evidence of the association of triglyceride and PC risk is still, and additional translation research is needed to confirm these findings. Although our results show association between lipid profiles and PC risk and aggressiveness, this data does not collect survival outcomes, thus further investigation should be needed. Despite these limitations, our study was a large study of 1,740 men from a single institution and is valuable as the only study that has examined the relationship between the lipid profile and risk, grade, and clinical stage of PC. Additionally, our data contained information on each patient’s diabetes status, statin-medication status, and HbA1c status, which are potential confounding factors that could affect patient misclassification if not evaluated.

## 5 Conclusions

Our findings suggest that high triglycerides are the most important component of the lipid profile to influence PC risk. Therefore, for patients who want lifetime monitoring of PC risk, it might be beneficial to focus on the triglyceride level. However, the protective effect of lowering the triglyceride level needs to be investigated through randomized-controlled trials in the future.

## Data availability statement

The raw data supporting the conclusions of this article will be made available by the authors, without undue reservation.

## Ethics statement

The studies involving human participants were reviewed and approved by Asan Medical Center Institutional Review Board. Written informed consent for participation was not required for this study in accordance with the national legislation and the institutional requirements.

## Author contributions

Jungyo Suh: Writing - Original Draft, Formal analysis, Teak Jun Shin: Writing - Original Draft Dalsan You: Data Curation, Writing - Review & Editing In Gab Jeong: Data Curation, Writing - Review & Editing Jun Hyuk Hong: Data Curation, Writing - Review & Editing Choung-Soo Kim: Data Curation, Writing - Review & Editing Hanjong Ahn: Conceptualization, Methodology, Writing - Review & Editing, Supervision
