# Supplementary material for: The association between serum lipid profile and the prostate cancer risk and aggressiveness
Source: Front Oncol. 2023 May 15;13:1113226. doi: 10.3389/fonc.2023.1113226 (PMC10225643; doi:10.3389/fonc.2023.1113226)
Supplement: Supplementary file 1 [file DataSheet_1.pdf]

Supplementary table 1. Multivariate quartile analysis for prostate cancer risk

A. Total cholesterol

	**1st Quartile**	**2nd Quartile**	**3rd Quartile**	**4th Quartile**	P trend
	<160	160-182	183-205	>205	
**PC risk**					
N = 720/1740	225/418	168/438	172/437	155/447	
Multivariable adjusted* OR (95% CI)	1.00 (Ref)	0.66 (0.44-0.99)	0.71 (0.42-1.19)	0.55 (0.26-1.18)	0.243

B. Triglycerides

	**1st Quartile**	**2nd Quartile**	**3rd Quartile**	**4th Quartile**	P trend
	<81	81-110	111-159	>159	
**PC risk**					
N = 720/1740	146/430	145/431	181/443	248/436	
Multivariable adjusted† OR (95% CI)	1.00 (Ref)	1.25 (0.88-1.76)	2.04 (1.41-2.95)	4.57 (2.94-7.11)	<0.001

* ORs are adjusted for Age, PSA, serum glucose, HbA1c, HTN, diabetes, LDL, HDL, and triglyceride.

† ORs are adjusted for Age, PSA, serum glucose, HbA1c, HTN, diabetes, total cholesterol, LDL and HDL

Supplementary table 2. General characteristics for pathologic Gleason grade (GG) group in prostate cancer patients

	**GG 1**	**GG 2-3**	**GG 4-5**	**p-value**
**Number of patients**	247	262	211	–
**Patients characteristics**				
Age (years)	64.2 (± 8.1)	66.6 (± 7.2)	68.5 (± 8.2)	< 0.001
BMI (Kg/m^2)	24.5 (± 2.6)	24.4 (± 2.7)	24.5 (± 3.2)	0.698
HTN	111 (44.9%)	128 (48.9%)	121 (57.3%)	0.009
Diabetes	47 (19.0%)	66 (21.0%)	65 (30.8%)	0.003
Serum glucose (mg/dL)	107.9 (± 22.6)	109.1 (± 26.9)	116.5 (± 29.0)	0.001
HbA1c	6.0 (± 1.0)	5.9 (± 0.8)	6.3 (± 1.2)	< 0.001
Statin comedication	26 (10.5%)	29 (11.1%)	31 (14.8%)	0.174
PSA (ng/mL)	5.4 (± 3.5)	7.0 (± 7.8)	97.4 (± 616.2)	0.004
Lipid profile				
Total cholesterol (mg/dL)	181.5 (± 38.4)	178.5 (± 35.5)	173.1 (± 33.6)	0.042
LDL (mg/dL)	113.8 (± 32.0)	112.7 (± 31.4)	107.9 (± 28.9)	0.103
HDL (mg/dL)	51.5 (± 13.7)	49.4 (± 12.8)	48.1 (± 13.0)	0.022
Triglyceride (mg/dL)	130.8 (± 62.5)	144.6 (± 76.9)	148.5 (± 78.6)	0.022

Supplementary table 3. Univariate quartile analysis for the association between serum triglyceride and pathologic Gleason grade group (GG) and clinical stage

	**1st Quartile**	**2nd Quartile**	**3rd Quartile**	**4th Quartile**	P trend
	<87	87-124	125-178	>178	
**GG 1 (Reference level)**					
N = 247/720	69/178	63/180	62/180	53/182	
**GG 2-3 versus GG 1**					
N = 262/720	63/178	63/180	71/180	65/182	
Univariate OR (95% CI)	1.00 (Ref)	1.10 (0.67-1.79)	1.25 (0.77-2.03)	1.34 (0.82-2.21)	0.646
**GG 4-5 versus GG 1**					
N = 211/720	46/178	54/180	47/180	64/182	
Univariate OR (95% CI)	1.00 (Ref)	1.29 (0.76-2.16)	1.14 (0.67-1.94)	1.81 (1.08-3.05)	0.136
**Organ-confined PC** (Reference level)					
N = 567/720	153/178	148/180	139/180	127/182	
**Locally advanced PC versus Organ confined**					

N = 112/720	15/178	24/180	34/180	39/182	
Univariate OR (95% CI)	1.00 (Ref)	1.65 (0.84-3.28)	2.50 (1.30-4.78)	3.13 (1.65-5.94)	0.003
**Metastatic PC versus Organ confined**					
N = 41/720	10/178	8/180	7/180	16/182	
Univariate OR (95% CI)	1.00 (Ref)	0.83 (0.32-2.15)	0.77 (0.29-2.08)	1.93 (0.85-4.40)	0.119

Supplementary table 4. General characteristics for the clinical stage in prostate cancer patients

	**Organ confined**	**Locally advanced**	**Metastatic**	**p-value**
**Number of patients**	567	112	41	–
**Patient characteristics**				
Age (years)	65.6 (± 7.8)	68.3 (± 7.5)	70.7 (± 9.1)	< 0.001
BMI (Kg/m^2)	24.4 (± 2.7)	25.0 (± 2.8)	23.9 (± 3.9)	0.052
HTN	272 (48.0%)	61 (54.5%)	27 (65.9%)	0.016
Diabetes	120 (21.2%)	31 (27.7%)	16 (39.0%)	0.004
Serum glucose (mg/dL)	109.3 (± 26.2)	114.8 (± 22.6)	121.4 (± 35.0)	0.004
HbA1c	6.0 (± 1.0)	6.2 (± 0.7)	6.9 (± 1.2)	< 0.001
Statin comedication	64 (11.3%)	16 (14.4%)	6 (14.6%)	0.311
PSA (ng/mL)	6.5 (± 9.1)	15.0 (± 16.7)	447.2 (± 1355.0)	< 0.001
Lipid profile				
Total cholesterol (mg/dL)	179.3 (± 36.5)	174.4 (± 33.4)	169.0 (± 37.0)	0.112
LDL (mg/dL)	112.5 (± 31.3)	110.4 (± 29.0)	104.1 (± 30.7)	0.219
HDL (mg/dL)	51.0 (± 13.4)	45.3 (± 11.2)	44.4 (± 11.8)	< 0.001

Triglyceride (mg/dL)	134.9 (± 68.2)	165.4 (± 87.6)	158.9 (± 80.4)	< 0.001
